# APADB: a database for alternative polyadenylation and microRNA regulation events

**DOI:** 10.1093/database/bau076

**Published:** 2014-07-21

**Authors:** Sören Müller, Lukas Rycak, Fabian Afonso-Grunz, Peter Winter, Adam M. Zawada, Ewa Damrath, Jessica Scheider, Juliane Schmäh, Ina Koch, Günter Kahl, Björn Rotter

**Affiliations:** ^1^Plant Molecular Biology, Molecular BioSciences, University of Frankfurt am Main, Marie-Curie-Street 9, D-60439 Frankfurt, Germany, ^2^GenXPro GmbH, Frankfurt Innovation Center Biotechnology, Altenhöferallee 3, D-60438 Frankfurt, Germany, ^3^Molecular Bioinformatics Group, Faculty of Computer Science and Mathematics, Cluster of Excellence Frankfurt “Macromolecular Complexes”, Institute of Computer Science, Robert-Mayer-Strasse 11-15, D-60325 Frankfurt am Main, Germany, ^4^Department of Internal Medicine IV; Saarland University Medical Center, Kirrberger Strasse, D-66421 Homburg/Saar, Germany, ^5^Experimental Neurology, Department of Neurology, Goethe University Medical School, Heinrich, Hoffmann Strasse 7, D-60528 Frankfurt am Main, Germany, ^6^Institute for Ecology, Evolution and Diversity, Aquatic Ecotoxicology, University of Frankfurt am Main, Max-von-Laue-Str. 13, D-60438 Frankfurt, Germany and ^7^Department of Pediatrics, University Hospital Schleswig-Holstein, Schwanenweg 20, D-24105 Kiel, Germany

## Abstract

Alternative polyadenylation (APA) is a widespread mechanism that contributes to the sophisticated dynamics of gene regulation. Approximately 50% of all protein-coding human genes harbor multiple polyadenylation (PA) sites; their selective and combinatorial use gives rise to transcript variants with differing length of their 3′ untranslated region (3′UTR). Shortened variants escape UTR-mediated regulation by microRNAs (miRNAs), especially in cancer, where global 3′UTR shortening accelerates disease progression, dedifferentiation and proliferation. Here we present APADB, a database of vertebrate PA sites determined by 3′ end sequencing, using massive analysis of complementary DNA ends. APADB provides (A)PA sites for coding and non-coding transcripts of human, mouse and chicken genes. For human and mouse, several tissue types, including different cancer specimens, are available. APADB records the loss of predicted miRNA binding sites and visualizes next-generation sequencing reads that support each PA site in a genome browser. The database tables can either be browsed according to organism and tissue or alternatively searched for a gene of interest. APADB is the largest database of APA in human, chicken and mouse. The stored information provides experimental evidence for thousands of PA sites and APA events. APADB combines 3′ end sequencing data with prediction algorithms of miRNA binding sites, allowing to further improve prediction algorithms. Current databases lack correct information about 3′UTR lengths, especially for chicken, and APADB provides necessary information to close this gap.

**Database URL:** http://tools.genxpro.net/apadb/

## Introduction

Translation and stability of messenger RNA (mRNA) is influenced by a multitude of factors, such as microRNAs (miRNAs) (1), long non-coding RNAs (2) and polyadenylation (PA) (3).

The addition of poly(A) tails to the 3′ end of RNAs in eukaryotic cells comprises a variety of mechanisms depending on the cellular localization (nucleus, cytoplasm or mitochondria) and the respective RNA. Nuclear PA of RNAs (especially of precursor mRNAs, pre-mRNAs) generated by eukaryotic RNA polymerase II occurs as a co-transcriptional process and depends on the presence of defined poly(A) signals in the nascent RNA (4). Poly(A) signals are recognized by several proteins, which synergistically lead to endonucleolytic cleavage of the nascent RNA, followed by addition of adenine residues to the newly formed 3′ end (PA site) of the upstream cleavage product *via* poly(A) polymerase. However, PA of RNAs transcribed by RNA polymerase II is not stringent. For example, 3′ end formation of histone pre-mRNAs involves cleavage only (5). Moreover, the poly(A) tails of mRNAs transcribed within mitochondria are added by the noncanonical poly(A) polymerase PAPD1 after endonucleolytic cleavage of the mitochondrial precursor RNA and are composed of ∼50 adenosine residues (6). In general, nuclear and cytosolic PA stabilizes the RNA and thereby delays its degradation (7). According to Chang and Tong, the same holds true for the addition of poly(A) tails to many, but not all mitochondrial mRNAs (6).

Alternative polyadenylation (APA) represents a common and critical regulatory mechanism in gene expression that involves the use of different PA sites for one and the same transcript. Distinct poly(A) signals can either be sequestered or exposed to generate RNA isoforms with different 3′ ends (8). These isoforms consequently harbor distinct coding sequences and/or 3′ untranslated regions (3′UTRs), which are causatively linked to altered function and stability or translation efficiency. Yoon and colleagues (9) underlined the role of differential mRNA PA site usage by showing that PA sites are not randomly distributed, but mainly flank regulatory signals, such as miRNA binding sites. Loss of recognition sites for RNA binding domains, a result of 3′UTR shortening by APA, has been reported for cancer cell lines (10). Global shortening of 3′UTRs *via* APA is associated with increased survival of cancer cells because many oncogenes escape miRNA regulation owing to a reduced availability of miRNA binding sites (11). In line with the dedifferentiated characteristics of cancer cells, a trend toward shortened 3′UTRs was identified in early mouse embryonic stem cells (12), and reprogramming of somatic to induced pluripotent stem cells involves general shortening of 3′UTRs (13). Regarding adipogenesis, APA ensures a modest but consistent elongation of 3′UTRs during differentiation, and thus allows for a fine-tuned transcript regulation *via* small non-coding RNAs (14). This underlines the importance of analyzing APA in combination with information about proven as well as predicted miRNA binding sites.

To date, databases with PA site annotations are sparse, and the available information is far from being complete. PolyADB2 (15) contains PA sites for human, mouse, rat, chicken and zebra fish, but the database is limited because complementary DNA (cDNA)/expressed sequence tag (EST) data are not completely available. Similar to polyADB2, PACDB (16) contains mRNA PA sites of several organisms based on only limited cDNA/EST data. In the meantime, next-generation sequencing (NGS) technologies rapidly advanced, and as a consequence, experimental evidence for particular PA site usage can readily be generated by 3′ end sequencing. Several tissue-specific APA events have been reported during the past year (17–19), but the respective data are not available as a public resource. AURA (20) represents a comprehensive and manually curated catalog of human UTRs and UTR regulatory annotations including PA sites, but the resource is limited to human and based on RNA sequencing data that does not explicitly focus on the 3′ end.

Here we present APADB, a user-friendly database designed to catalog all PA sites in human, mouse and chicken. PA sites are identified by massive analysis of cDNA ends (MACE), a high-throughput NGS-based 3′ end sequencing technique, in combination with an optimized data analysis pipeline. APADB can be screened for several tissues of an organism, and the user can either examine all identified PA sites or search for specific PA sites within a gene of interest. The supporting read alignments for each site can be visualized in a genome browser with additional information about respective miRNA binding sites, gene structure and an according view of sites from polyADB2. APADB improves current annotations from the RefSeq database and provides evidence for (1) PA sites of several genes encoding pre-miRNAs as well as (2) several lost miRNA binding sites that should be taken into account in miRNA–mRNA interaction prediction tools. Databases of predicted miRNA–mRNA interactions are based on the assumption that the full length of the 3′UTR is always expressed, which is only rarely the case. Approximately half of all human genes harbor more than one PA site (21), and analysis of APA sites by MACE even extends this number to 64, 52 and 73% of all mRNAs (in human, mouse and chicken, respectively).

## Methods

**Figure 1.**
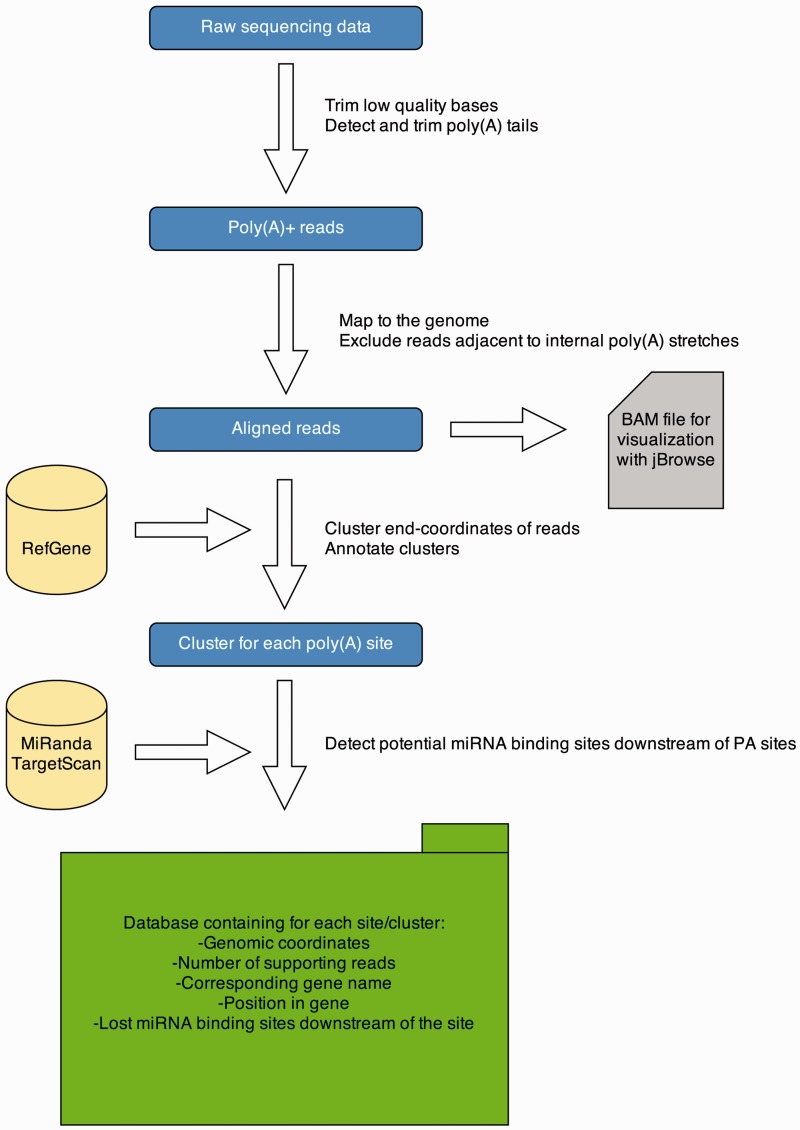
APADB analysis pipeline. Analysis of a MACE library starts with quality trimming and *de novo* poly(A) tail detection. Poly(A) tail positive reads are mapped to the genome under consideration. Reads adjacent to genomic poly(A) stretches are discarded and accepted alignments are stored in BAM format for visualization *via* jBrowse. Mapped reads are summarized to clusters and annotated. Potentially lost miRNA binding sites downstream of the cluster are detected using TargetScan and miRanda predictions. All information is stored in an SQL database, containing information about the location of the PA site, the number of supporting reads and potentially lost miRNA binding sites.

To detect PA sites in several tissues from different organisms, we applied a 3′ end sequencing technique, namely, MACE. These data are analyzed with a Python pipeline, and results are presented on a Web-platform. An overview of the data analysis pipeline is given in Figure 1. The pipeline is implemented in Python 2.7 and R 3.0.1. A detailed description of each processing step is provided in the subsequent sections.

### Tissue samples

PA sites were characterized in seven different human tissue types from adults: kidney, pancreas, peripheral blood mononuclear cells (PBMCs), whole blood, pancreatic ductal adenocarcinoma (PDAC), papillary renal cell carcinoma (PRCC) and acute lymphoblastic leukemia (ALL). RNA from decapitated *Gallus gallus* embryos (embryonic day 6) served for identification of chicken PA sites, whereas mouse data were derived from muscle and liver tissue. The samples were sequenced at different depths, ranging from 669 283 polyadenylated reads in human PDAC to 43 415 110 reads in the chicken library. Table 1 summarizes the statistics for all samples.

**Table 1.** Summary of generated data

	Raw reads	Poly(A) + reads	PA clusters	Intersection with PolyADB2
Human				
Whole blood	349 733 599	14 221 576	34 693	20 044
Kidney	7 292 11	1 371 093	10 903	9710
Pancreas	51 550 704	1 141 836	4771	4413
PBMCs	107 809 349	5 164 979	19 945	14 744
ALL	16 236 500	908 744	8369	6783
PDAC	51 621 788	669 283	7573	6986
PRCC	15 450 055	2 126 524	17 544	14 451
Chicken				
Decapitated chicken	284 325 540	43 415 110	23 039	2654
Mouse				
Liver	40 993 932	7 002 487	21 119	13 293
Muscle	43 467 565	7 556 000	21 869	13 529

Raw reads are filtered, mapped to the genome under consideration, filtered for internal priming artifacts, clustered and annotated to known genes.

### Massive analysis of cDNA ends

MACE represents an NGS-based 3′ end sequencing protocol developed by GenXpro GmbH (22). The fact that MACE reads are always derived from the outermost 3′ end of mRNA allows the identification of PA sites on a genome-wide scale, and without prior knowledge of respective gene sequences or PA signals. Additionally, MACE implies the use of GenXPros TrueQuant technology that allows polymerase chain reaction (PCR)-bias-free quantification of sequencing reads. Briefly, total RNA is reverse-transcribed using an anchored and biotinylated oligo(dT) primer that preferentially hybridizes to the proximal end of poly(A) tails. To minimize inadvertent internal priming of homopolymeric adenine stretches, hybridization is performed at 50 C (hot priming) for identification of PA sites in mouse and chicken, whereas cDNA synthesis of human tissues was initialized at room temperature. Reverse-transcribed cDNA is fragmented by sonication and subsequently bound to a streptavidin matrix. Unbound fragments are discarded and biotinylated fragments released from the matrix for ligation of the sequencing adaptor. The adaptor-ligated fragments are amplified *via* PCR and finally sequenced on the Illumina HiSeq2000 platform.

### Quality trimming, *de novo* poly(A) tail detection and clipping

To remove low-quality bases from the start/end of reads, all bases detected as a low-quality segment (FASTQ Sanger quality score <16) indicated by the special Q2 score, are clipped from both sites of reads. This increases mapping accuracy and prevents false-negative results in the poly(A) tail detection.

In comparison with other methods (23) MACE does not guarantee that the generated sequences end directly adjacent to the poly(A) tail owing to random fragmentation into 50–500 base pair (bp) fragments. Consequently, only a subset of fragments shorter than 100 bps can be used for a clear poly(A) tail assignment.

Therefore, we implemented a *de novo* poly(A) tail detection script to identify reads containing noncanonical poly(A) tails and to ensure the usage of all sequenced bases starting immediately after the poly(A) tail of the RNA molecule they were derived from. A sliding window with a size of five bases is progressively moved toward the 5′ end starting from the 3′ end of each read. In each step, the presence of at least four adenines in the window is verified (one base serves as a margin for sequencing errors). When less than four adenines are present, the current position of the window is evaluated. If it has successfully moved seven or more steps away from the 3′ end, the read is considered as poly(A) tail positive. The poly(A) tail of these reads is trimmed off, and the trimmed sequences are passed on to the next step of the pipeline.

### Read mapping

The poly(A) trimmed reads are mapped to the respective genome using Novoalign (http://novocraft.com). Only uniquely mapped reads are taken into account for subsequent analysis, controlled by ‘-r none’ and without soft clipping (‘-o FULLNW’).

### Bioinformatical exclusion of inadvertent internal priming of homopolymeric adenine stretches

Nam and colleagues demonstrated that generation of truncated cDNAs *via* inadvertent internal priming of homopolymeric adenine stretches during oligo(dT)-based reverse transcription is a frequent event (24). Even a stretch of eight adenines in 10 bps within a given RNA sequence can already be sufficient for annealing of the oligo(dT) primer at room temperature. Without particular consideration, these internally primed RNAs would result in the detection of false-positive poly(A) sites. Apart from minimizing this effect in most of our libraries *via* hot priming during reverse transcription, we used a *de novo* detection script for identification of genomic homopolymeric adenine stretches that can give rise to truncated cDNAs. First, genomic stretches are determined by mapping *AAAAAAAAAA* to the respective genome with bowtie (25), allowing two errors (‘-v 2, -r all’), transforming alignments to BED format and merging overlapping intervals with BEDtools (26). Subsequently, uniquely mapped poly(A) tail positive reads are screened for adjacent homopolymeric adenine stretches, as defined in the BED file. The respective reads containing such a stretch are considered as false positive and excluded from further analysis.

### Clustering of mapped reads

To identify clusters of reads representing one PA site, we implemented a 1D clustering algorithm: For each strand on each chromosome, mapping end-coordinates of all poly(A) tail trimmed reads (the poly(A) tail starts directly after this position) are sorted numerically. Clusters are represented by the median end-coordinate of its members. As most PA sites cluster in a 24-bp window, but the distance between the most upstream and the most downstream cleavage sites could reach up to 30–40 bps (21), a cluster is extended by the next end-coordinate from the sorted list, if it is at most 25 bps away from the representative. Otherwise, the cluster is closed, and the genomic coordinates together with the number of reads falling in the cluster, the median and the mode are reported in BED format. Afterward, a new cluster is opened and extended until the described distance criterion is violated. This strategy guarantees that most clusters will have a size between 1 and 25 bps, but does not restrict clusters to a maximal size for special cases where the cleavage site wobbles by >25 bps.

### Annotation of clusters

Respective RefSeq tracks from the UCSC table browser (UCSCTB) are used for the annotation of identified clusters (27). For each gene, the genomic coordinates of exons, introns, 5/3′UTRs and extensions are stored in BED format and intersected with the mapping coordinates of the clusters. In the case of multiple features, overlapping one cluster, the following hierarchical structure is applied: ‘3′UTR>exon>intron>5′UTR>extension’. Only the highest feature from the hierarchy is reported. Extensions represent regions downstream of an annotated 3′UTR that do not overlap any other feature. This procedure allows for extension of known 3′UTRs, and can thus improve the current RefSeq tracks of 3′UTRs, especially in the case of chicken, where most extensions are reported.

### Prediction of potentially lost miRNA binding sites

For human and mouse tissue, the genomic locations of predicted miRNA binding sites [TargetScan 6.2 (28)] within 3′UTRs of mRNAs are extracted from the UCSCTB. For chicken, FASTA sequences of all 3′UTRs from the UCSCTB are used to predict potential interactions with mature chicken miRNAs available from miRBase v20 (29) using miRanda (30). MiRNA binding sites downstream of a given PA site within the same gene are reported to be lost for case of usage of this respective PA site.

### Motif search for poly(A) signals

**Figure 2.**
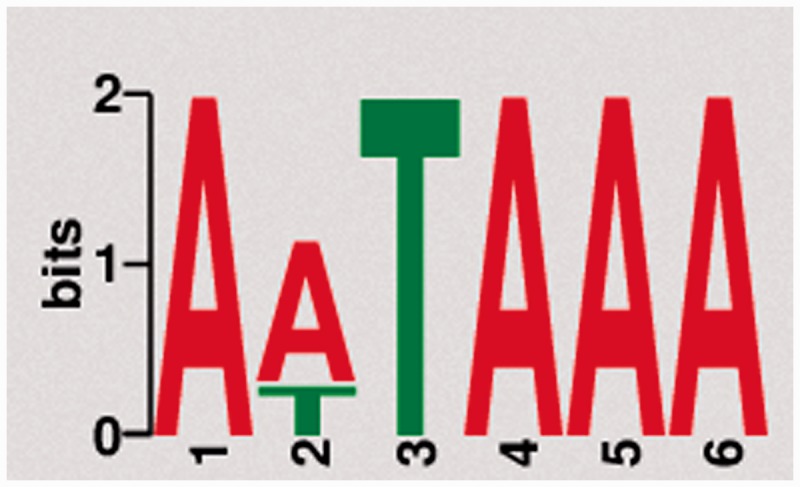
Motif discovery. The most frequent and significantly enriched motif discovered upstream of PA sites from pooled human tissue samples.

Naturally, PA signals are located proximal to their respective PA site. The canonical AAUAAA recognition sequence, for example, is typically located within 20–30 bps of the respective PA site (31). To test for previously unknown motifs and to validate the reliability of our data with respect to the known canonical poly(A) signals, we used DREME, a tool for motif discovery in large sequencing data sets (32). Sequences onto 50 bps upstream of the detected PA sites were extracted in FASTA format *via* BEDtools and subsequently used for motif discovery. The most frequent motif (AWUAAA) in the human whole blood sample is exemplary shown in Figure 2. The motif is found upstream of 23 045 of 34 693 PA clusters (66.4%). In total, we found 18 significantly (e-value < 0.05) enriched motifs with a length of 5–8 bps (Supplementary Table S1). The fact that the mentioned motif exhibits the highest frequency among all significantly enriched motifs across all samples and organisms is in line with the expected prevalence of canonical poly(A) signals and affirms the integrity of the present data set.

#### Web server implementation

**Figure 3.**
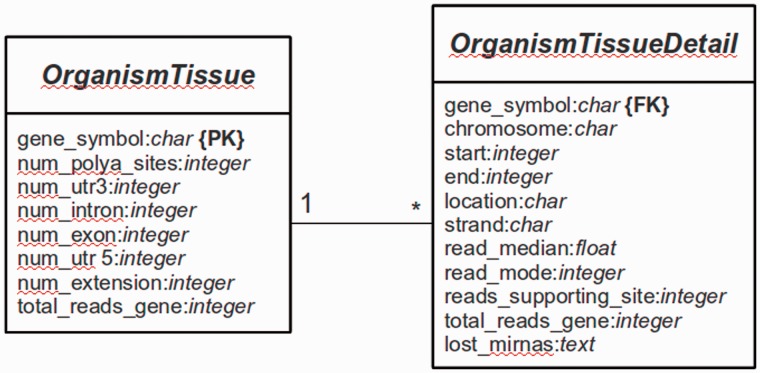
Database structure. The entity relationship diagram describes the structure of the database.

The main platform is implemented in Python 2.7 using the Web framework Django (version 1.4.9) (http://www.djangoproject.com). The database is managed with PostgreSQL (version 9.1). Django is a framework that follows the Model-View-Controller (MVC) paradigm, with the advantage to separate the site design from the programming logic. The whole Web platform is implemented in Django, with a base system as the core of the platform and several services [e.g. omiRas (33), APADB] as build-in modules. For APADB, basically two prototypes (in MVC terminology called ‘Models’) are used to describe the table structures for each organism and tissue (see Figure 3). Both tables are connected through a PK<–>FK (PK: primary key; FK: foreign key) relationship by the field ‘gene_symbol’. Django abstracts the SQL layer through an object-relational mapper (ORM) that acts as a dynamic database-access application programming interface (API). The ORM allows to perform database queries in a simpler way through Python internal objects, avoiding raw SQL-based code. Fetched data are processed by the Django app ‘django-tables2’ (https://github.com/bradleyayers/django-tables2) that allows to create HTML-based table structures (with sorting options) directly from models and queries. The JavaScript-based genome browser jBrowse (version 1.10.9) (34) is integrated into the Django system within a design template.

## Results

**Figure 4.**
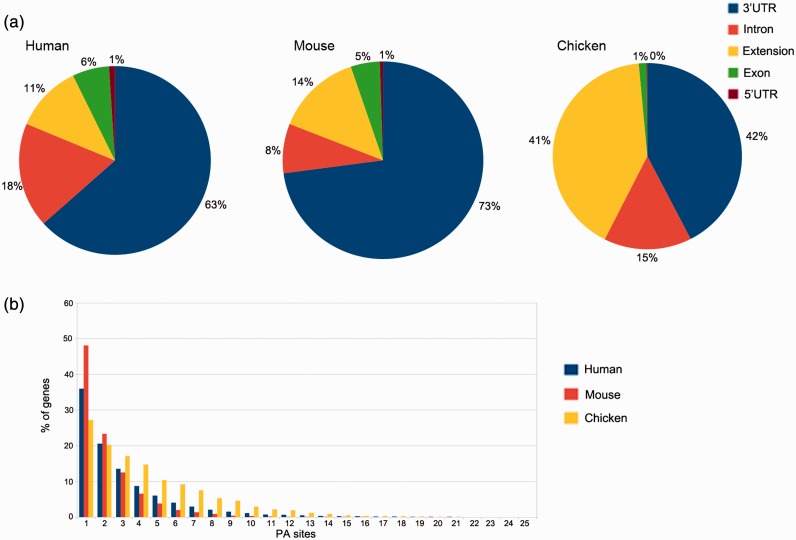
PA site distributions. (**a**) The distribution of PA sites across different genomic regions for each organism, as defined by the RefSeq annotation track. The 3′UTRs harbor the most PA sites, whereas relatively few sites are located in 5′UTRs. Extensions are defined as PA sites located < 3 kb downstream of an annotated RefSeq 3′UTR. (**b**) Number of genes and PA sites for all the analyzed organisms. The majority of genes harbors only one PA site independently of the respective organism.

*In silico* pooling of all reads from human samples resulted in 50 718 PA sites within 15 717 identified genes. As expected, the majority of these sites is located in the annotated RefSeq 3′UTR (32 141) or up to 3 kb downstream (5809). Taken together, coding exons and introns harbor 12 266 PA sites, and only 502 are located in the 5′UTR (Figure 4a). The pooled mouse sample displays a similar distribution, except for intronic regions that display 10% fewer and 3′UTR regions that harbor 10% more PA sites. The distribution for chicken, however, differs from the other two organisms. A large proportion of clusters is located up to 3 kb downstream of the annotated RefSeq 3′UTR (see extension). These extensions indicate incomplete annotations of 3′UTRs in the current RefSeq database. Only one PA site was present in 36% of human genes, 42% of the genes contain between two and four PA sites, 17% between five and 10 sites and 5% contain >10 and up to 30 sites (Figure 4b), which is consistent with previously reported numbers (21).

The median equals two PA sites per gene, and on average, a gene comprises 3.2 PA sites. These numbers differ for mouse and especially for chicken samples. While 48% of the genes comprise a single PA site in pooled mouse tissue, the number is reduced to 27% in chicken. The relatively low amount of sequenced reads from humans and especially mice (only two samples) is one of the factors that may contribute to these differences. Another reason is the relatively low diversity of tissue types for the two mammals, as the chicken embryos were almost used entirely for library preparation.

**Figure 5.**
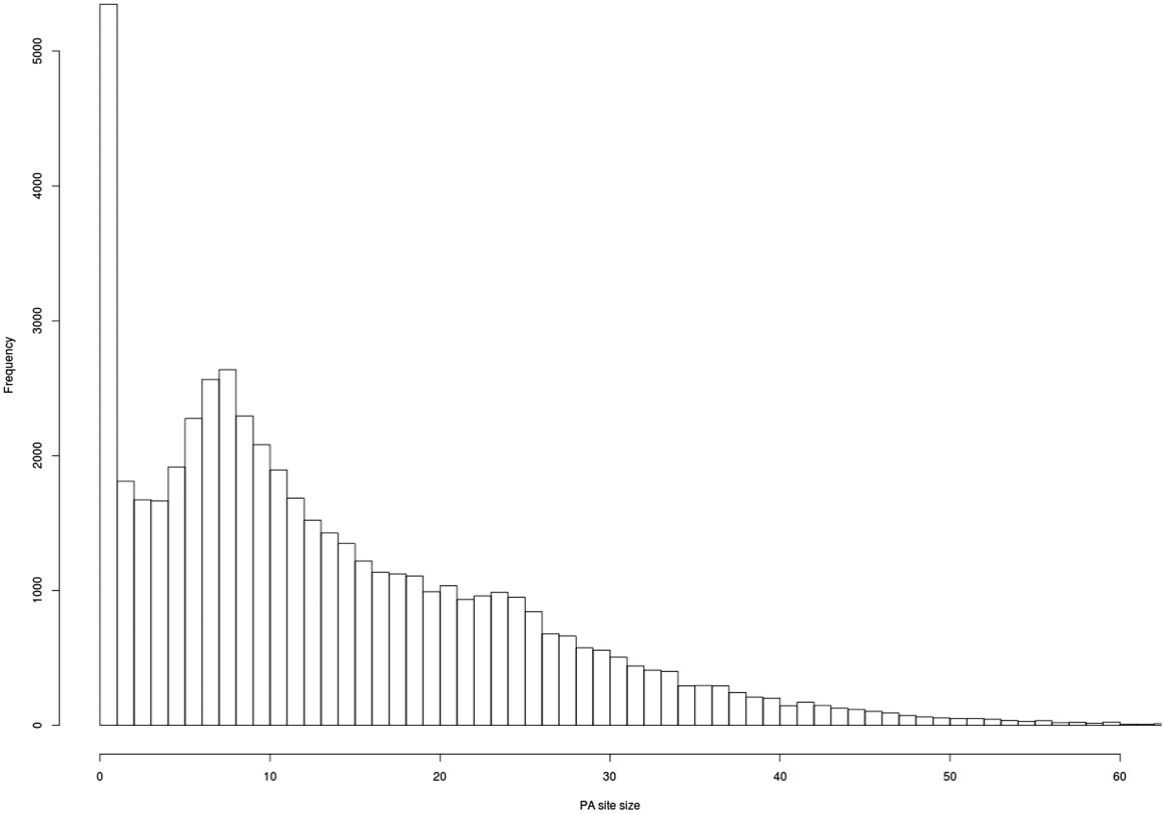
Evaluation of PA site cluster size. A histogram shows the frequency of PA clusters depending on their size. Most PA clusters exhibit a size of 1–25 bps.

As expected, most of the clusters show a clear PA site at a single nucleotide (Figure 5), and most of the PA site clusters have a size of 1–25 bps.

### Comparison with polyADB2

Currently, polyADB2 represents the most comprehensive database of PA sites. APADB extends the information about PA sites for chicken from 5830 entries in polyADB2 to 23 039 PA sites. A total of 25 966 human PA sites from polyADB2 are supported by MACE reads. In all, 4683 PA sites from polyaDB2 are adjacent to internal poly(A) stretches and excluded from our analysis. Additionally, we find 18 714 new sites within RefSeq 3′UTRs, not currently reported in polyADB2. The high percentage of overlap between both databases for the human samples underlines the reliability, and the identification of a large number of novel PA sites in human and especially in chicken by MACE affirms the potential of this technique for the characterization of APA. Nevertheless, 28 720 PA sites from PolyADB2 currently not annotated in APADB underline the need of frequent updates with new human tissue samples.

The sample-specific intersections of PA sites detected by MACE and PA sites in polyADB2 are given in Table 1. For human kidney, 89% of PA clusters can be found in polyADB2, while only 58% of the clusters are also found in polyADB2 when compared with the whole blood sample. These differences reflect the differing sequencing depths. Rare PA variants require a high-sequencing depth for detection, as in the whole blood sample, which is sequenced ∼30 times deeper than the kidney sample.

### Comparison with 3P-seq NGS data

The 3P-seq mouse liver data set published by Nam and colleagues (35) was downloaded from gene expression omnibus (GSM1268948) to compare the PA sites identified by ADADB with those detected by an NGS-based method that is not affected by internal priming. The coordinates in the BED file provided by Nam and co-workers were lifted from the mm9 to mm10 mouse genome using the LiftOver tool from UCSC genome browser (http://genome.ucsc.edu/cgi-bin/hgLiftOver). After extraction of PA sites supported by at least five reads (in the following referred to as high-confidence sites—HCS) the BED file comprised 22 447 PA sites with 3 784 516 supporting reads.

**Figure 6.**
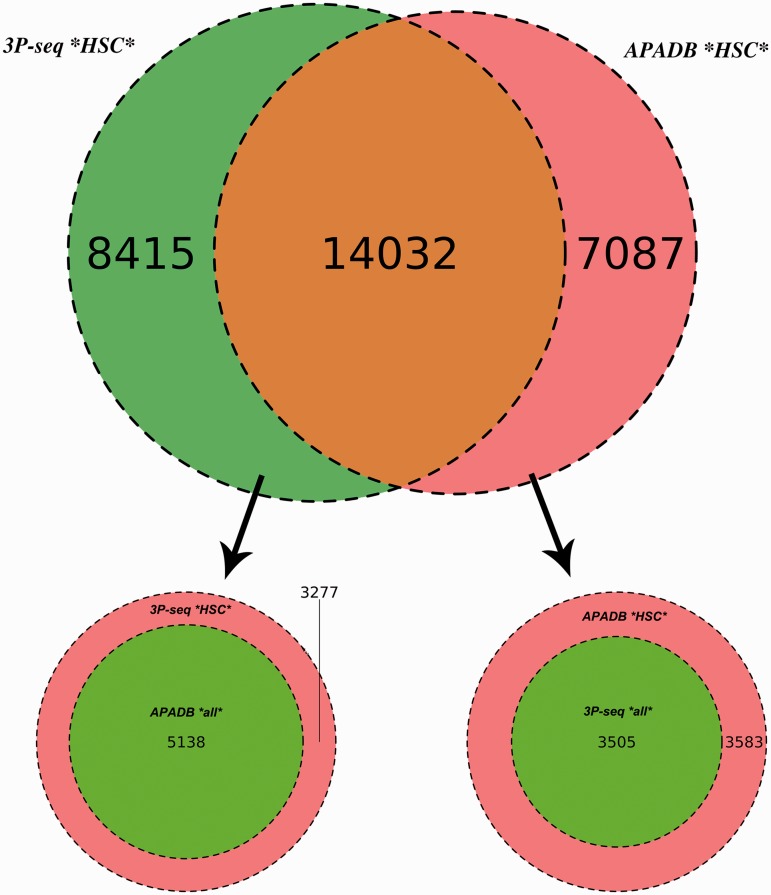
Comparison of APADB and 3P-seq. The Venn diagram on top shows the HCS (supported by more than four NGS reads) for mouse liver samples detected exclusively by 3P-Seq, by both methods and sites only listed in APADB. Of 8415 HCS exclusive to 3P-seq, 5138 are contained in APADB without a threshold on read depth (bottom left). The diagram on the bottom right indicates that of 7087 HCS exclusive to APADP, 3505 are contained in 3P-seq data without a minimum read cutoff.

A similar amount of HCS (21 119) and supporting reads (3 681 375) is listed in APADB for mouse liver. The results of the comparison are illustrated in Figure 6.

In total, 14 032 HCS are detected by both methods, while 7087 and 8415 HCS (APADB and 3P-seq, respectively) are only present in one of the data sets. The comparison of APADB’s HCS to all sites given by 3P-seq (without any threshold of supporting read numbers) reveals that 17 537 PA sites are supported by both methods, whereas 3583 sites (17% of all APADB entries) are exclusively listed in APADB. Of these, 1701 clusters comprise only 5–9 supporting reads. Conversely, the comparison of the HCS from 3P-seq with all the identified sites from APADB reveals that 19 170 of all 3P-Seq HCS are detected by both methods. Of the remaining 3277 (15% of all 3P-seq HCS) clusters, 2295 are only supported by 5–9 reads.

The large overlap between PA sites detected by both methods (83% of MACE HCS and 85% of 3P-seq HCS sites) underlines the reliability of MACE for PA site detection. Additionally, the similar percentages of sites that are only detected by one of the methods point to an efficient exclusion of false-positive PA sites arising from internal priming. Assuming a large number of false-positive sites in APADB because of internal priming, the fraction of exclusive sites in APADB would clearly exceed those sites exclusive to 3P-seq. With similar percentages of exclusive sites detected by each method, the reason for this exclusive detection is likely linked to the biological variance of gene expression and the technical variance introduced during library preparation and sequencing that especially affects lowly expressed variants.

### Graphical user interface and case study

The functions and capabilities of APADB are exemplary demonstrated by an analysis of the identified PA sites from the KRAS oncogene. KRAS is upregulated in different cancer types, and post-transcriptional regulation of KRAS *via* interaction with miR-216a/217 was reported *in vitro* (36).

**Figure 7.**
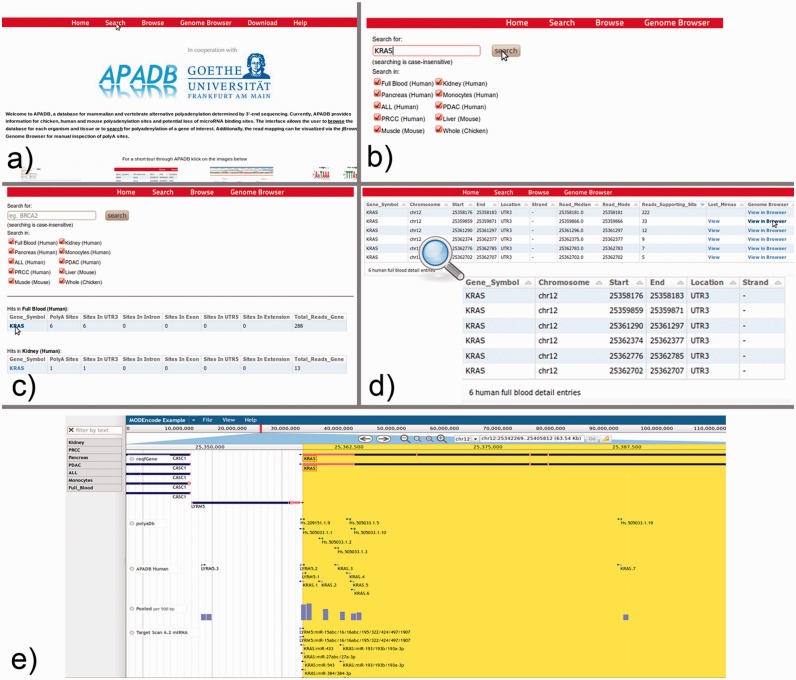
KRAS serves as an example for the use of the APADB Web interface to find and visualize PA sites. The search page (**b**) can be accessed from the main page (**a**) *via* the ‘Search’ link. The search page allows for entering a HUGO name of interest, and subsequently the respective results are listed for each organism and tissue (**c**). Detailed information about the PA sites is called up by selection of the gene name in a specific tissue type (**d**). An alignment of reads corresponding to a given PA site can then be visualized in jBrowse (**e**) together with the corresponding clusters in APADB, miRNA binding sites and sites contained in polyADB2.

From the APADB main page, where the core functions of the Web interface are introduced, the database search can be initialized *via* the ‘Search’ button from the top menu (Figure 7a). Within the search page (Figure 7b), the user can enter the HUGO name of a gene of interest and select the tissue samples that are to be scanned. In case of KRAS, PA sites are available for several human, as well as chicken and mouse tissues. An excerpt of the identified PA sites of KRAS is shown in Figure 7c, and selection of the gene symbol from the tissue of interest calls up a detailed view of respective PA sites (Figure 7d). The detailed view presents information on the genomic locations of PA sites, the number of supporting reads, the median and mode of read ends in these clusters and links to a list of lost miRNA binding sites in the case of non-canonical PA site usage. Additionally, alignments of MACE reads to the corresponding genome are available *via* a link to the genome browser.

For KRAS in human whole blood (six PA sites, all in the 3′UTR), the most frequent PA site is located directly at the distal end of the known 3′UTR, and consequently no miRNA binding sites are lost. The other variants with shorter 3′UTRs, however, loose between 16 and 36 potential miRNA binding sites, and among these are the experimentally validated binding sites of miR-216a and miR-217. The lack of binding sites for miRNAs can be visually inspected in the genome browser, which merges information of the RefSeq genes, entries from APADB, polyADB2, predicted miRNA binding sites from TargetScan/miRanda and read alignments from the respective tissues. In the case of the second most frequent PA site of KRAS, 16 potentially lost miRNA binding sites can be seen upstream of the respective cluster of supporting MACE reads for this PA site (Figure 7e). These (and even shorter) isoforms also are no longer responsive to the validated regulation of KRAS by miR-216a/217. Post-transcriptional regulation of KRAS through these miRNAs is consequently only relevant for a subset of KRAS mRNAs, explicitly those with the longest 3′UTR.

Finally, if the user is not looking for a specific target gene, but rather interested in an overall picture of APA in certain tissues, a corresponding overview is provided *via* the browse link on the main page. After selection of a sample, the user can browse through the (A)PA events of each expressed gene from the sample, or sort the PA sites according to, for example, number of supporting reads, number of PA sites in exons or introns and others. The visualization of MACE reads in the jBrowse genome browser can directly be accessed by the Genome Browser link. Subsequent to selection of an organism, the user is directed to the genome of his choice, and alignments from different samples can be dragged into the interface for visualization.

## Discussion

We present a comprehensive database of (A)PA sites in human, mouse and chicken based on an optimized NGS-coupled 3′ end sequencing protocol, namely, MACE. We provide additional experimental support for PA sites reported in polyADB2, and identify novel, previously not annotated PA sites, especially for chicken tissue, where up to 41% of the genes display a much longer 3′UTR than currently known. The user friendly and intuitive Web interface can be used to browse the database by genes, by organism as well as tissue and to visualize the NGS reads corresponding to PA sites in a genome browser. We combine PA sequencing data with a bioinformatical prediction of miRNA binding sites that are lost in alternatively polyadenylated transcripts owing to 3′UTR shortening. The unique combination of features and the comprehensive amount of compiled data for human, mouse and chicken recommends APADB as a useful resource for improvement of current 3′UTR annotations, miRNA–mRNA interaction prediction algorithms, as well as tissue-specific miRNA regulation.

## Supplementary data

Supplementary data are available at *Database* Online.
